# Ketamine: Linking NMDA receptor hypofunction, gamma oscillations and psychosis (commentary on Qin et al., 2022)

**DOI:** 10.1111/ejn.15872

**Published:** 2022-12-07

**Authors:** Thomas J. Reilly

**Affiliations:** ^1^ Department of Psychiatry University of Oxford, Warneford Hospital Oxford UK

Ketamine acts primarily through non‐competitive NMDA receptor antagonism but has myriad downstream effects. Previously best known as an anaesthetic, analgesic or substance of abuse, it is now part of psychiatry's toolkit as an anti‐depressant (Price et al., 2022). Ketamine is also useful as a model of psychosis, reproducing symptoms and allowing examination of corresponding brain states.

It was another NMDA receptor antagonist, phencyclidine, that led to the NMDA hypofunction model of schizophrenia (Olney et al., 1999). A key component of this model is acute NMDA antagonism causing a state in healthy volunteers with many similarities to schizophrenia. It has stood the test of time and is supported by recent neuroimaging studies, showing lower hippocampal NMDA receptor availability in patients with first episode psychosis compared with healthy controls (Beck et al., 2021).

Ketamine's effect on gamma band oscillations potentially links NMDA receptor hypofunction with symptoms and cognitive dysfunction in psychosis. Gamma oscillations arise from an inhibition and rebound excitation cycle mediated by parvalbumin positive GABAergic interneurons (Bartos et al., 2007). Notably, dendritic NMDA receptors on these interneurons are critical to establish synchronous neuronal assemblies (Rupert & Shea, 2022). This synchronisation underlies cortical computation and enables numerous cognitive processes such as memory and sensory processing (Ward, 2003).

Cumulating evidence implicates gamma band abnormalities in psychosis. Reduced gamma power evoked by auditory click trains (the auditory steady state response) is a robust finding in schizophrenia (Thun et al., 2016). By contrast, resting‐state gamma power is increased, which may reflect more baseline ‘noise’ in the brain (White & Siegel, 2016). Dysfunction in gamma oscillations is proposed to result in the higher cognitive deficits and behavioural disorganisation associated with schizophrenia (Lesh et al., 2011).

In the recent study by Qin et al. in the *European Journal of Neuroscience*, the authors examined the effect of ketamine on oscillatory activity and sensory processing in a rodent model, making use of more invasive measures of oscillations than is normally possible in human subjects. In keeping with the abnormalities associated with psychosis, ketamine resulted in increased baseline gamma power but decreased power in response to a sensory stimulation. The authors suggest this decreased the signal‐to‐noise ratio and disrupted sensory processing. In support, the authors found a decrease in coherence between the ventral posteromedial nucleus of the thalamus and Layer 6 of the related somatosensory cortex. They also found increased multistate entropy in this system, indicating an increased randomness of the signal.

Like all models, ketamine as a psychotomimetic has its limitations. Although ketamine reproduces psychosis symptoms in both healthy controls and patients with schizophrenia, positive symptoms are significantly greater than negative symptoms (Beck et al., 2020). Whether it can still be considered a psychotomimetic in an anaesthetised rodent is open to debate. Certainly, we are unable to model the higher cognitive processes inherent to humans and at the heart of schizophrenia.

Furthermore, there is discrepancy in the findings of acute ketamine administration in healthy controls compared with first episode psychosis and chronic schizophrenia groups. Grent‐'t‐Jong et al. (2018) showed that while ketamine reproduced symptoms of psychosis, its effect on gamma power and connectivity (induced visually by a high contrast circular moving grating) were opposite to abnormalities seen in the patient groups.

These limitations notwithstanding, Qin et al. further our understanding of how NMDA receptor antagonism through ketamine administration perturbs gamma oscillations and associated sensory processing. Doing so provides another link between NMDA hypofunction and dysfunction in circuits associated with psychosis.

## CONFLICT OF INTEREST

T.J.R. reports no competing interests.

### PEER REVIEW

The peer review history for this article is available at https://publons.com/publon/10.1111/ejn.15872.
